# The rising prevalence of obesity: part A: impact on public health

**DOI:** 10.1097/IJ9.0000000000000017

**Published:** 2017-06-22

**Authors:** Maliha Agha, Riaz Agha

**Affiliations:** a Department of Primary Care and Public Health, King’s College London; b Department of Plastic Surgery, Guy’s and St. Thomas’ NHS Foundation Trust, London, UK

**Keywords:** Obesity, Overweight, Public health

## Abstract

Excessive fat accumulation in the body may impair health leading to a significant long-term health consequences including the development of diabetes, coronary heart disease, and osteoarthritis as well as increasing the risk of developing certain cancers and influencing their outcomes. England has some of the worst figures and trends in obesity compared with the rest of the Europe. In the majority of European countries the trend has increased from 10% to 40% in the last 10 years, whereas in England prevalence has more than doubled. This article outlines the public health impact of rising obesity levels.

## Introduction

**Figure 1:**
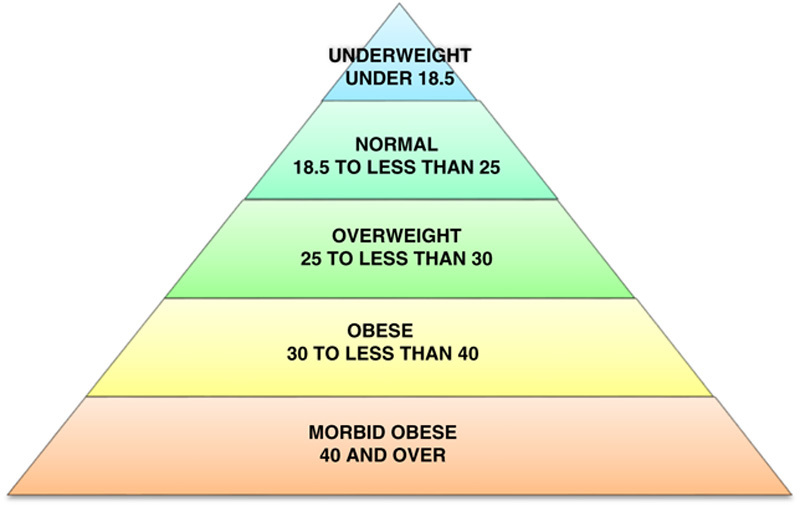
Body mass index definitions (kg/m^2^). Source: Centres for Disease Control and Prevention, 2011.

Obesity is an abnormal accumulation of body fat (usually 20% above the normal ideal body weight) to the extent that it may have an adverse effect on health^1^. It is a chronic disorder, officially classified as a disease (ICD-10 E66.0) in 1990 and defined as a body mass index (BMI) of 30 kg/m^2^ or more (**Fig.** **1**)^2^,^3^. Obesity is a rapidly growing public health problem affecting an increasing number of countries worldwide because of its prevalence, costs, and health effects^4^.

**Figure 2:**
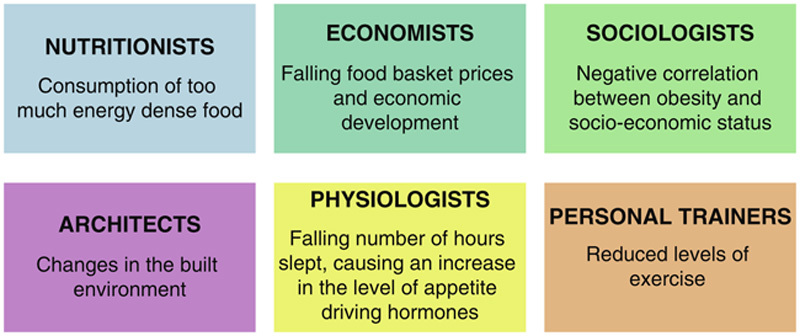
Causes of obesity.

Compared with the medical definition of obesity^5^, the public health explanation places emphasis on the preventive and causative factors. Effective treatment requires identification and modification of these etiological vectors^6^. However, the causes are often complex or not well understood. In addition, every discipline has dimensionalized the problem along different lines (**Fig.** **2**)^7^.

## Obesity a growing public health problem

In 1995, there were an estimated 200 million obese adults worldwide. However, by the year 2000 the number had grown to 300 million and has continued to increase since then^8^.

The trends for increasing obesity are broadly repeated throughout Western Europe and a similar rise has been observed in the United States. In 1986, 1 in 200 adults in America were morbidly obese; by 2004, the figure was 1 in 50. Currently, 1 in 5 adults are morbidly obese in America^9^.

The rates of obesity have similarly increased in the United Kingdom since the 1980s, this rise is projected to continue. The 2008 Health Survey for England report indicated that 1 in 4 adults were obese^10^.

Since 1991 the prevalence of obesity has increased by 65% in men, and 25% in women. It was estimated that in 2010, England contained 6.6 million obese men (33% of the population) and 5.9 million obese women (28% of the population)^11^. The proportion of men predicted to be obese was greater than the proportion of women^12^. It has been estimated that on current trends, by 2050, 60% of males and 50% of females will be obese^13^.

**Figure 3:**
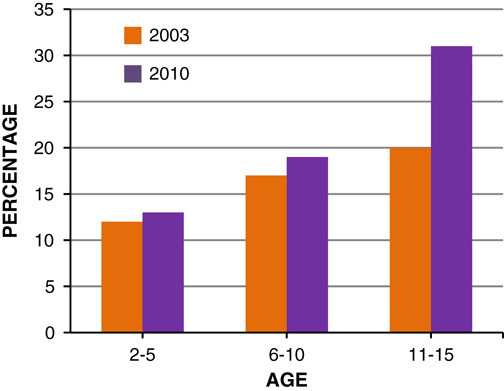
Prevalence of boys overweight and obese in 2003 and 2010, by age.

**Figure 4:**
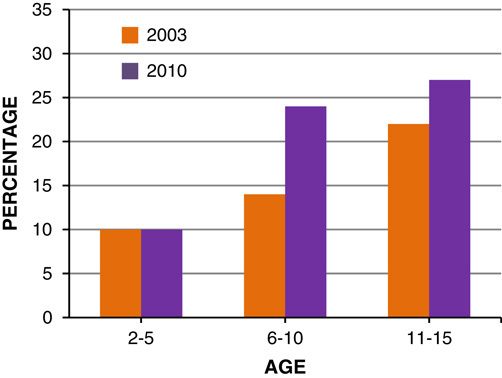
Prevalence of girls overweight and obese in 2003 and 2010, by age.

The current trend shows that around 8% of 1–2-year-old obese children will become obese adults, and 80% of 10–14-year olds will become obese adults^14^. In 2003, approximately 750,000 boys and 676,000 girls were obese in England. If the same trend is applied to mid-2010 population, it is estimated that 792,321 (19% of population) boys and 910,630 (22% of population) girls were obese in 2010 (**Figs.** **3**, **4**)^8^.

**Figure 5:**
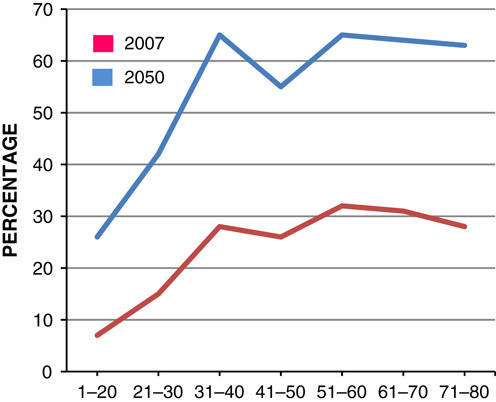
Estimated percentage of male obese at 2007 and 2050.

**Figure 6:**
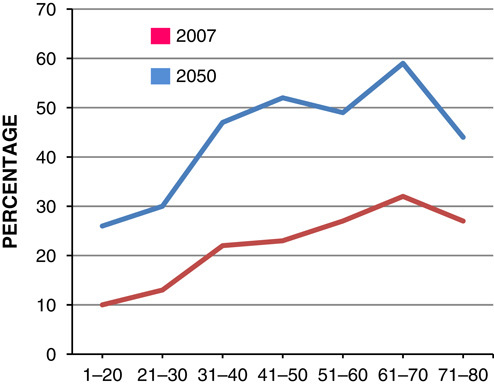
Estimated percentage of female obese at 2007 and 2050.

Evidence suggests that by 2050, among children aged 6–10 years, the number of obesity among boys will be greater than girls with an estimate of 50% of boys being obese by 2050 (**Figs.** **5**, **6**)^10^.

**Figure 7:**
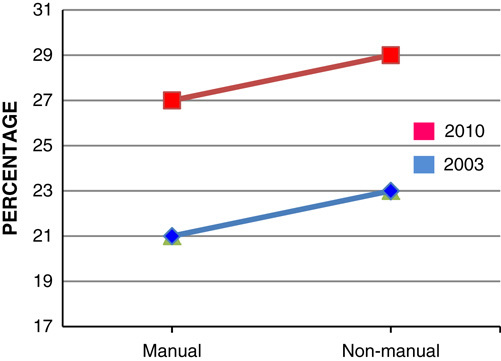
Prevalence of men obese in England in 2003 and 2010 by social class.

**Figure 8:**
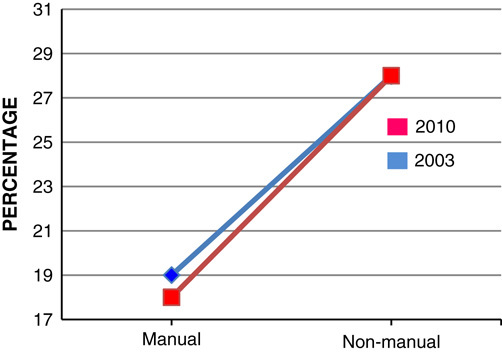
Prevalence of females obese in England in 2003 and 2010 by social class.

Adults working in unskilled manual profession are over 4 times more likely to be classified as morbidly obese compared with those in professional employments, with women showing a larger difference than men (**Figs.** **7**, **8**)^15^. It is estimated that by 2050, 15% of females belonging to social class I and 62% for the females belonging to social class V will be obese. However, the predicted difference is lower for male adults, 52% of males belonging to social class I will be obese compared with 60% of social class V males^15^.

**Figure 9:**
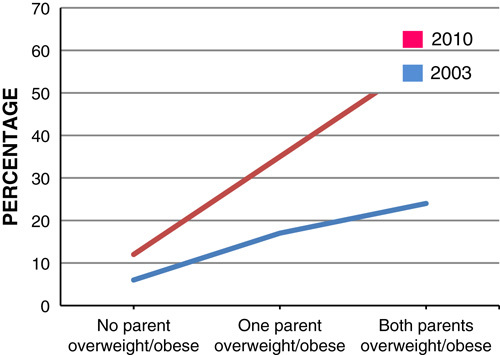
Prevalence of boys overweight and obese in 2003 and 2010, by parental body mass index status.

**Figure 10:**
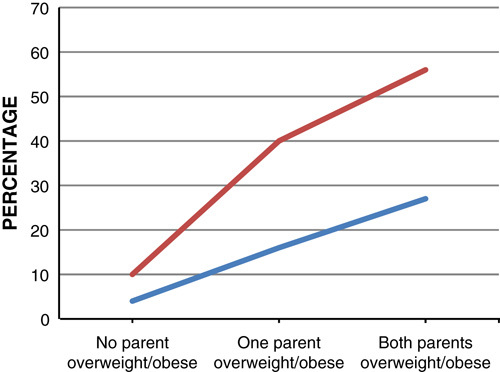
Prevalence of girls overweight and obese in 2003 and 2010, by parental body mass index status.

The prevalence of obesity is higher in children who live in households where both the parents are overweight or obese. In 2010, around 415,844 boys and 320,727 girls were estimated to be obese by their parental obesity status^11^. Therefore, boys may be more affected by their parental weight than girls (**Figs.** **9**, **10**). If 1 parent is obese the odds ratio of a 1–2-year old being an obese adult is 30% more than nonobese and it increases by 17-fold for a 15–17-year old. The increased prevalence of obesity in childhood is likely to continue to adult life making them more susceptible to illness^11^.

**Figure 11:**
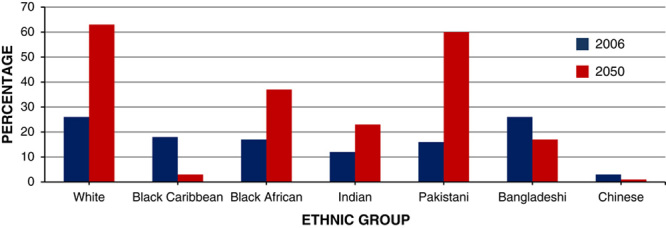
Predicted percentage of male population obese at 2006 and 2050, by ethnic group.

**Figure 12:**
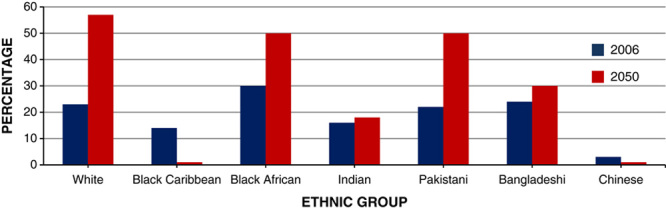
Predicted percentage of female population obese at 2006 and 2050, by ethnic group.

Ethnicity is one of the major determinants in obesity trends. It is estimated that by 2050, people belonging to Black Caribbean and Chinese ethnic backgrounds will be less obese than the current value. Asian children are also 4 times more likely to be obese than white children (**Figs.** **11**, **12**)^16^.

**Figure 13:**
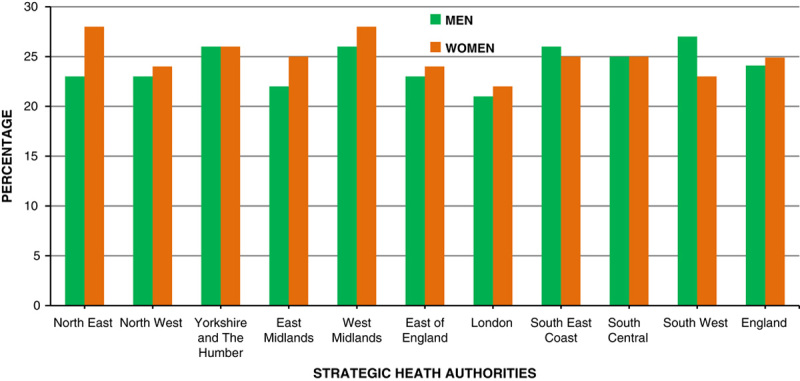
Obesity prevalence rate 2008/2009.

Evidence suggests that a significant difference exists between regions in England. By 2050, estimates suggest that 70% of women in Yorkshire and Humberside will be obese, whereas in other regions such as the south-west of England it will be 7% (**Fig.** **13**).

## The impact of increased prevalence of obesity on public health

### Life expectancy

Overall life expectancy is expected to rise 8 years for males and 7 years for females by the mid-21st century^17^. However, the increased prevalence of obesity may have long-term ramifications for this trend among young adults and children. If the prevalence of obesity increases as predicted, females will lose around a fifth of a year and males around a third by the middle of the 21st century^18^. Therefore, the rising trend for obesity will significantly affect the health of the population as well their contribution to the economy and society^4^. Concomitantly, health care costs and the rates of diseases/conditions caused and influenced by obesity will increase. These are further downward drivers life expectancy^19^.

### Employment

Obesity has become stigmatized, and discrimination presents significant challenges in education, health care, and employment. There is weak evidence that the IQ of overweight children is lower than those of normal weight^20^–^22^. This results in stress, psychological distress, depression, social isolation, low self-esteem, and poor body image. Therefore, due to the increased prevalence of obesity, unemployment may increase and appropriately qualified obese individuals may stay unemployed due to discrimination or fear of it in the workplace^23^.

### Quality of life

Evidence suggests that obesity is associated with shortness of breath, back pain, reduced mobility, poor quality of life, and increased psychological and social burden^24^.

In the older population, obesity causes cartilage degradation and increases the risk of physical and cognitive disability^25^,^26^. These are major risk factors associated with institutionalization, greater health care costs, dependency, utilization of health care services, poor health outcomes, reduced exercise capacity, and mortality^27^.

Sarcopenia is a condition of age-related loss of muscle mass and strength and is a common problem in old age. Evidence suggests that older people with sarcopenia and obesity have worse physical function than those with sarcopenia alone. Such effects on disability and quality of life may not just be additive but synergistic^28^–^30^.

### Obesity-associated diseases

**Figure 14:**
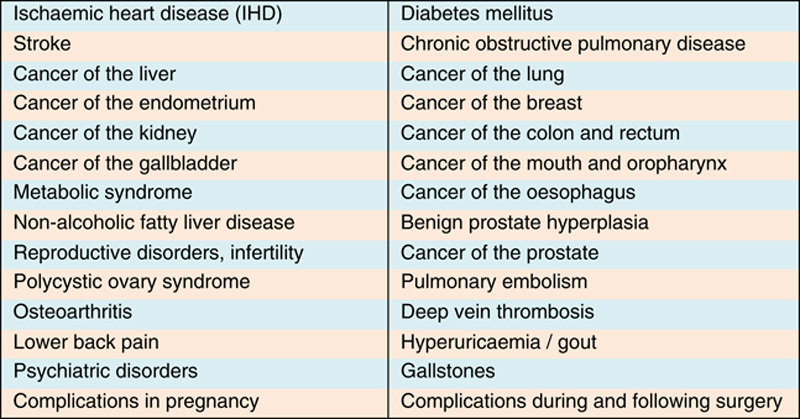
Diseases associate with obesity.

In the United Kingdom, it is estimated that each year 9000 premature deaths are due to obesity, accounting for 8.7% of all deaths^31^. It is highly associated with other health problems and diseases leading to disability and in many cases death (**Fig.** **14**)^32^. Because obesity rates vary among different ethnicities, the diseases associated with it may result in health inequalities (**Figs.** **13**, **14**)^33^. Among adults the most common chronic conditions associated with obesity include: arthritis, hypertension, heart disease, stroke, cancer, and diabetes^34^. We have talked about the impact on preconceptual and pregnant women elsewhere^35^.

The result of these associations is that the relative risk of death increases with BMI in a nonlinear manner (**Table** **1**)^36^.

**Table 1:**
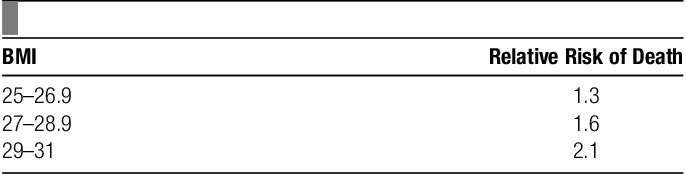
Classification of body mass index (BMI) and relative risk of death.

#### Diabetes

Diabetes associated with obesity, is a massive and a growing threat to public health^37^. The susceptibility of diabetes is 80 times greater among obese adults than nonobese. Professor Sir George Alberti, President of the International Diabetes Federation, has stated that those with a BMI of 35 have “a 92-fold increase in the risk of diabetes” compared with those with a BMI of 22^38^. The consequences associated with diabetes include: congestive heart disease, blindness, kidney failure, stroke, osteoarthritis, leg ulcers, and limb amputation. It was projected that there were 3 million (15%) diabetes patients living in the United Kingdom in 2010^39^ and this is expected to increase to 70% in 2035. If the obesity trend continues the same way, by 2025 a quarter of the health budget will be spent on diabetes^40^.

#### Coronary heart disease (CHD)

CHD susceptibility increases 2–3 times more in obese adults. It is estimated that obesity accounts for 5% of CHD deaths in men and 6% in women^40^. If the obesity trend continues, it will increase from 10% in 2010 to 20% in 2035^41^.

#### Cancer

Obese adults are 40% more likely to die from cancer than nonobese. Post menopausal obese women also have a higher risk of developing breast and endometrial cancers^41^. Although both obese men and women have a higher risk of developing cancers of the esophagous, colon, rectum, kidney, pancreas, thyroid, and gallbladder, cancer due to obesity accounts for 14% of deaths in men and 20% in women^42^. Obesity has been considered as the most important avoidable cause of cancer in nonsmokers, as proposed by Professor Julian Peto^43^. A recent survey showed that, although obesity is the main preventable risk factor after tobacco, only 3% of the UK population knew that cancer is associated with obesity^44^.

#### Osteoarthritis

Osteoarthritis associated with obesity affects the knees, hips, and the lower back by placing extra pressure on these joints and wearing away the cartilage^44^. This results in back, knee, and hip pain and therefore leading to difficulty in walking. More than 11 million working days are lost each year due to obesity in Britain and increasing prevalence means losing more days causing greater economic disruption^45^.

#### Emotional and psychological damage

Emotional and psychological problems due to obesity is a massive public health problem^46^. This results in lower self-esteem, anxiety, clinical depression, and suicidal attempts in extreme cases. Obese people are 3–4 times more likely to be depressed than nonobese^47^. Obese women are 37% more likely to commit suicide^48^. The emotional damage caused by obesity results in binge-eating, low confidence, social isolation, and humiliation^49^. Professor Hubert Lacey, from the Royal College of Psychiatrists said that obesity causes depression rather than depression causing obesity^15^.

### Economic costs

The increase in the prevalence of obesity will cause a significant impact on the National Health Service (NHS) budget. Other costs include: absence from work, morbidity not treated in the health service, and the affect on quality of life (**Table** **2**).

**Table 2:**
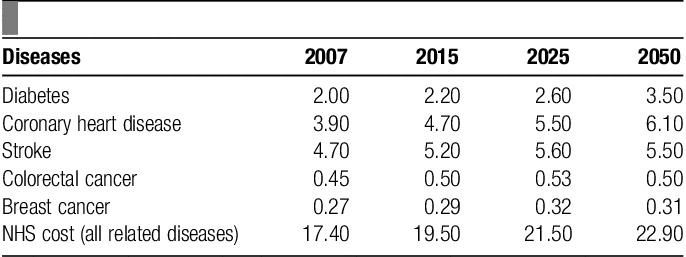
Estimated future NHS costs of diseases related to body mass index, 2007–2050 (£billion/y).

In 1998, the direct cost of treating obesity and its associated consequences was £480 million (1.5% of NHS expenditure) and indirect costs such as loss of earnings and premature mortality was £2.1 billion, giving an overall total of £2.58 billion. It had been projected that the total figure would rise to £3.6 billion in 2010^50^. However, due to the unexpected increases in the prevalence of obesity, NHS and drug costs from 1998 to 2010 has increased to £6.6–7.4 billion per year by the Clerk’s Department Scrutiny Unit^51^.

The health care costs associated with obesity among adults is higher among the middle aged^52^. As a consequence of increased cost and downward pressures on budgets, there may be increases in the cost of medicines, health care services, and taxes to compensate for the loss.

## Conclusions

The gloomier picture of the likely threat that the increased prevalence of obesity possesses is concerning. If the trend continues unabated, in future decades the sight of overweight and obese individuals will be greater than the normal weight population on the streets of the United Kingdom. Ultimately, this will result in increase in the number of amputees, blind people, cancer patients, and the demand for kidney dialysis will increase subsequently. The positive trends of fighting heart disease (mainly due to reduced smoking) in the recent decades will be reversed. De facto, the increased prevalence of childhood obesity is so serious that as a consequence, this will be the first generation in which children will die before their parents in significant numbers^52^. Therefore, obesity is likely to supersede tobacco as the biggest cause of premature death. It is the responsibility of many government departments besides the Department of Health to tackle this amplifying issue^53^.
